# Structural Characterization of Ettringite Synthesized in the Presence of Arsenate(V) and Chromate(VI) Ions

**DOI:** 10.3390/molecules31183305

**Published:** 2026-09-17

**Authors:** Magdalena Król, Witold Jastrzębski, Włodzimierz Mozgawa

**Affiliations:** Faculty of Materials Science and Ceramics, AGH University of Krakow, 30 Mickiewicza Av., 30-059 Kraków, Poland; witjas@agh.edu.pl (W.J.); mozgawa@agh.edu.pl (W.M.)

**Keywords:** ettringite, oxyanions, structural studies, vibrational spectroscopy

## Abstract

Ettringite (Ca_6_[Al(OH)_6_]_2_(SO_4_)_3_⋅26H_2_O) is an important hydration product involved in the immobilization of oxyanion contaminants in cementitious systems; however, the structural effects of arsenate(V) and chromate(VI) incorporation remain poorly understood. This study investigates the influence of both oxyanions on the formation and structure of ettringite-based materials using X-ray diffraction (XRD) with Rietveld refinement and Fourier-transform infrared spectroscopy (FT-IR). Ettringite remained the dominant crystalline phase in all samples, with no detectable secondary crystalline compounds. Chromate incorporation caused only minor changes in lattice parameters, whereas arsenate addition resulted in a decrease in unit cell volume, mainly due to contraction along the c axis. FT-IR analysis confirmed preservation of the ettringite framework and revealed local structural modifications, particularly for arsenate-bearing samples. The results indicate that the presence of both oxyanions is associated with structural changes in ettringite-bearing materials, with arsenate causing a stronger structural response within the investigated compositional range.

## 1. Introduction

Portland cement is the most widely used construction material worldwide, and its hydration processes lead to the formation of key binding phases that determine the properties of the cementitious matrix [1,2]. In the context of the rapid development of the metallurgical and energy industries, which generate enormous quantities of industrial solid wastes containing heavy metals, such as fly ash, blast furnace slag, and electroplating sludge, cement-based solidification/stabilization (S/S) technologies have become an essential tool for environmental protection [3,4]. The main hydration products of cement, namely AFt (aluminate–ferrite–trisulfate; ettringite) and AFm (aluminate–ferrite–monosulfate) phases, play a fundamental role in the chemical immobilization of toxic oxyanions such as chromates and arsenates [4].

**Figure 1:**
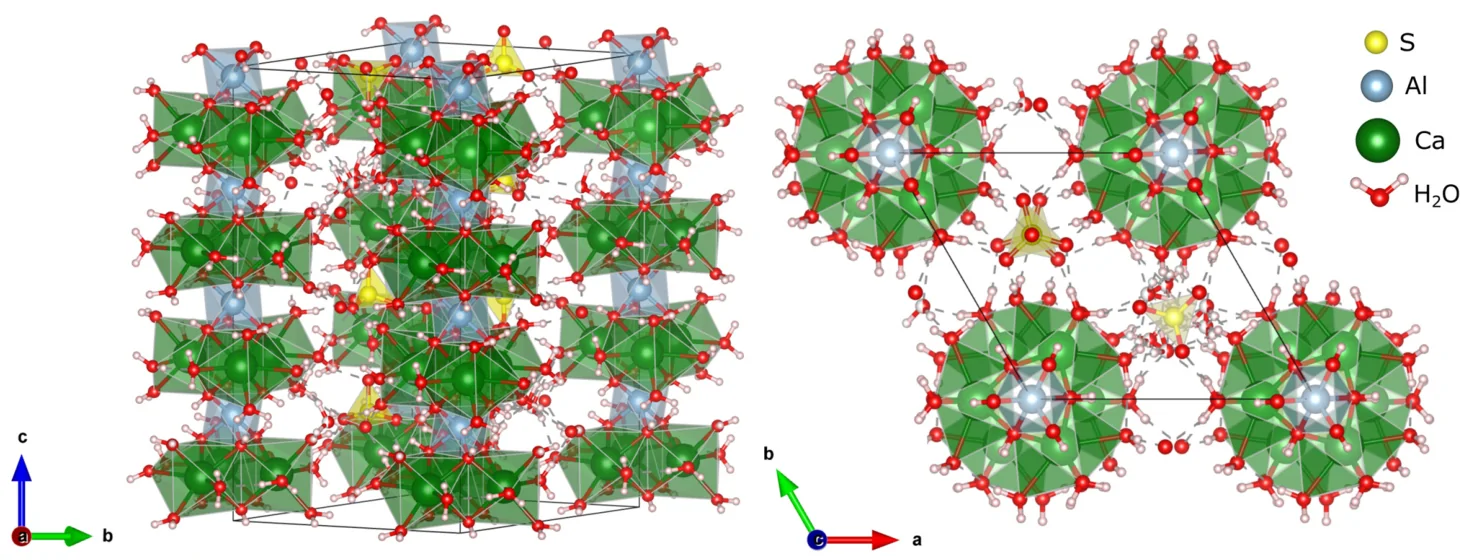
Crystal structure of ettringite showing the Ca–Al hydroxide columns and the channels occupied by sulfate ions and water molecules. Created using Vesta software (version 3) [14].

Ettringite (Ca_6_[Al(OH)_6_]_2_(SO_4_)_3_⋅26H_2_O) is characterized by a unique crystal structure composed of positively charged columns [Ca_6_[Al(OH)_6_]_2_⋅24H_2_O]^6+^ and intercolumn channels occupied by sulfate ions and water molecules [1,5,6]. A schematic representation of the crystal structure of ettringite is shown in Figure 1.

The presence of these channels enables the incorporation of various tetrahedral oxyanions through substitution of sulfate species [2,4,7], making ettringite one of the most important cement hydration products responsible for the immobilization of hazardous contaminants. In contrast, AFm phases possess a layered structure with positively charged main layers, [Ca_4_Al_2_(OH)_12_]^2+^, in which anionic species can be stabilized within the interlayer space, contributing to the retention of selected contaminants [4].

In natural mineralogy, the ettringite group encompasses a wide variety of minerals (e.g., charlesite, thaumasite) where sulfate is extensively replaced by carbonate or chromate species [8,9]. The natural mineral siwaqaite represents a Cr(VI)-bearing analogue of ettringite, in which CrO_4_^2−^ occupies the anion-bearing channels [10]. The flexible channel structure of these AFt phases allows accommodating both isovalent (e.g., SO_4_^2−^ ↔ CrO_4_^2−^) and heterovalent (e.g., SO_4_^2−^ ↔ HAsO_4_^3−^) substitution pathways, thereby influencing the stability and lattice expansion/contraction of the framework [7,8].

The ability of ettringite to incorporate hazardous oxyanions has attracted considerable attention in recent years due to its potential application in the S/S of contaminated wastes [2,4,5]. Numerous studies have demonstrated that chromate and arsenate species can be immobilized through incorporation into AFt and AFm phases formed during cement hydration [2,6,7,8,9,10,11]. The efficiency of this process depends on several factors, including pH [2,5], the concentration of competing ions [5,11], the availability of sulfate [11], and the stability of the hydration products under environmental conditions [12,13].

In cementitious systems, chromium is predominantly present in the hexavalent oxidation state, Cr(VI), which is characterized by high mobility, high solubility, and significant toxicity, making its effective immobilization an important environmental challenge. Chromate ions (CrO_4_^2−^) can be incorporated into the structures of both AFt and AFm phases through isovalent substitution for sulfate ions [11,15,16]. Under reducing conditions, a fraction of Cr(VI) may also be converted to the less mobile Cr(III) species, which can subsequently substitute for Al^3+^ within the octahedral sites of calcium aluminate hydrates, further enhancing chromium retention.

Arsenic is another highly toxic and carcinogenic element commonly encountered in industrial wastes. Under the highly alkaline conditions prevailing in cementitious systems, arsenic occurs predominantly as arsenate species and can be immobilized within the channels of the ettringite structure [5]. Experimental solubility studies combined with density functional theory (DFT) calculations have demonstrated that hydrogen arsenate (HAsO_4_^2−^) is the thermodynamically preferred species incorporated into ettringite, providing a stable mechanism for arsenic retention in hydrated cement systems [5].

Although the immobilization of chromate and arsenate by cement hydration products has been extensively investigated [17], the mechanisms governing their incorporation into the crystal structure of ettringite remain incompletely understood. In particular, the structural response of ettringite to the incorporation of isovalent chromate(VI) and heterovalent arsenate(V), including changes in lattice parameters, crystallinity, and crystal morphology, has received considerably less attention than contaminant removal efficiency or leaching behaviour [4,7]. Understanding these mechanisms at the molecular level is essential for optimizing hazardous waste stabilization processes and for assessing the long-term durability of cementitious materials exposed to aggressive corrosive environments [7,17].

The incorporation of oxyanions into ettringite may occur through different structural mechanisms depending on the charge and chemical characteristics of the substituting species. For chromate(VI), the replacement of SO_4_^2−^ by CrO_4_^2−^ can occur through isovalent anionic substitution without requiring additional charge compensation [16]. In contrast, incorporation of AsO_4_^3−^ requires charge compensation; previous studies have suggested that hydrogen arsenate, HAsO_4_^2−^, may be preferentially incorporated into the ettringite channels, allowing substitution for sulfate while maintaining local charge balance [4,5,7]. Such incorporation may also modify the arrangement of structural water and hydrogen-bonding interactions within the channels [7]. Therefore, oxyanion incorporation may involve not only anionic substitution but also associated changes in charge compensation and the local hydration environment.

Therefore, this study aimed to investigate the influence of arsenate(V) and chromate(VI) ions on the formation and structural characteristics of synthetic ettringite. The synthesized materials were comprehensively characterized using X-ray fluorescence (XRF), scanning electron microscopy (SEM), X-ray diffraction (XRD), and Fourier-transform infrared (FT-IR) spectroscopy. Particular attention was paid to elucidate the structural response of ettringite to the incorporation of both oxyanions.

## 2. Results

The synthesized ettringite-based materials were first characterized in terms of their chemical composition and morphology to evaluate the influence of arsenate(V) and chromate(VI) incorporation on the formation of the ettringite phase. Samples synthesized in the presence of increasing chromate(VI) and arsenate(V) concentrations were labelled as 1Cr–5Cr and 1As–5As, respectively. X-ray fluorescence (XRF) spectroscopy was applied to determine the elemental composition of the obtained solids; scanning electron microscopy (SEM) was used to examine their morphology and microstructural features, while X-ray diffraction (XRD) and Fourier-transform infrared spectroscopy (FT-IR) were employed to identify the crystalline phases and investigate structural changes associated with chromate(VI) and arsenate(V) incorporation into the ettringite framework.

The elemental compositions determined by XRF for the reference ettringite and oxyanion-containing samples are presented in Table 1. The analysis confirmed the presence of the characteristic elements of the ettringite framework, including Ca, Al, and S. At the same time, As- and Cr-bearing samples additionally contained the corresponding oxyanion-forming elements. The total chromium content, expressed as Cr_2_O_3_ equivalent increased from 0.02 wt.% in sample 1Cr to 1.31 wt.% in sample 5Cr, while the total arsenic content, expressed as As_2_O_5_, increased from 0.13 wt.% in sample 1As to 9.01 wt.% in sample 5As. The variation in elemental composition reflects the different synthesis conditions and provides information on the retention of arsenate and chromate species within the obtained solid phases.

**Figure 2:**
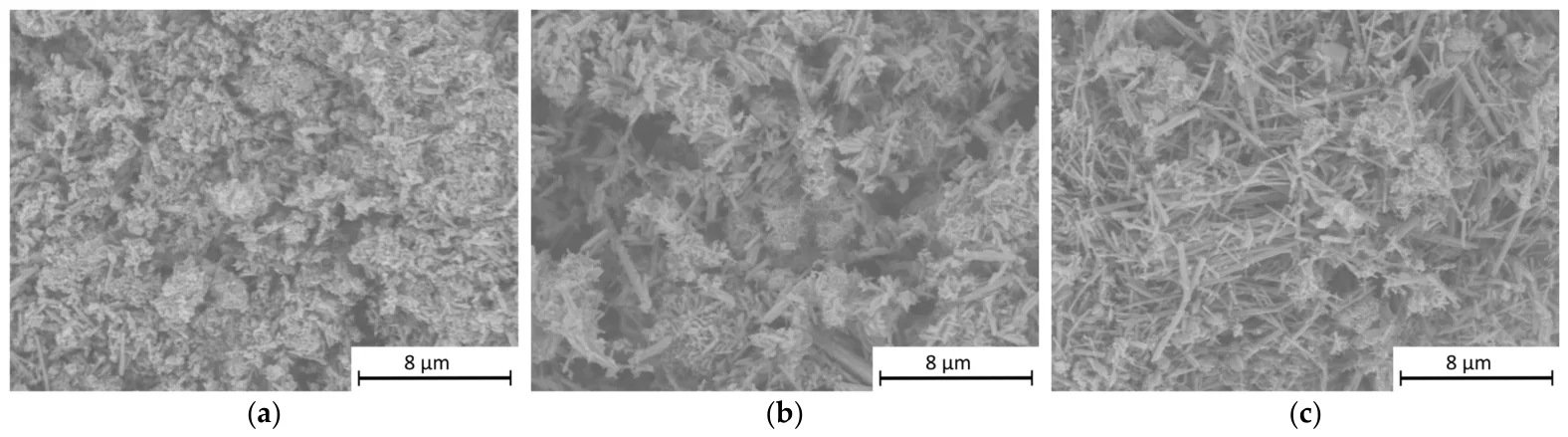
SEM micrographs of (**a**) reference ettringite, (**b**) chromate(VI)-containing ettringite (5Cr), and (**c**) arsenate(V)-containing ettringite (5As).

The morphology of the synthesized materials was examined by SEM, and representative micrographs are presented in Figure 2. The reference ettringite sample exhibited the characteristic acicular morphology of ettringite, consisting of densely packed needle-like crystals forming compact aggregates. Similar crystal morphology was observed for both oxyanion-bearing samples, confirming that the formation of the characteristic ettringite habit was preserved in the presence of chromate(VI) and arsenate(V). Nevertheless, differences in the degree of crystal aggregation were observed. The chromate-containing sample (5Cr) exhibited a more open aggregate structure in which individual acicular crystals were more readily distinguished than in the reference sample. The arsenate-containing sample (5As) displayed the most pronounced development of elongated needle-like crystals, forming a relatively loose network with a lower degree of aggregation than the reference ettringite.

**Table 1:** Oxide composition of the synthesized ettringite samples determined by XRF analysis (wt.%, reported as conventional oxide equivalents).

Sample	Name	CaO	Al_2_O_3_	SO_3_	As_2_O_5_	Cr_2_O_3_	Others
reference ettringite	ref.	60.26	8.94	30.03	0.00	0.00	99.22
	1Cr	58.06	9.02	32.07	0.00	0.02	99.17
	2Cr	58.32	9.68	31.08	0.00	0.08	99.16
chromate(VI)-bearing	3Cr	57.75	10.11	30.81	0.00	0.22	98.89
	4Cr	58.80	8.97	30.75	0.00	0.64	99.15
	5Cr	56.01	9.80	31.64	0.00	1.31	98.74
	1As	58.38	9.78	30.50	0.13	0.00	98.88
	2As	57.28	10.16	30.98	0.71	0.00	99.13
arsenate(V)-bearing	3As	58.68	9.23	29.47	1.18	0.00	98.55
	4As	55.48	10.00	29.36	4.34	0.00	99.17
	5As	55.72	8.29	25.99	9.01	0.00	99.00

**Figure 3:**
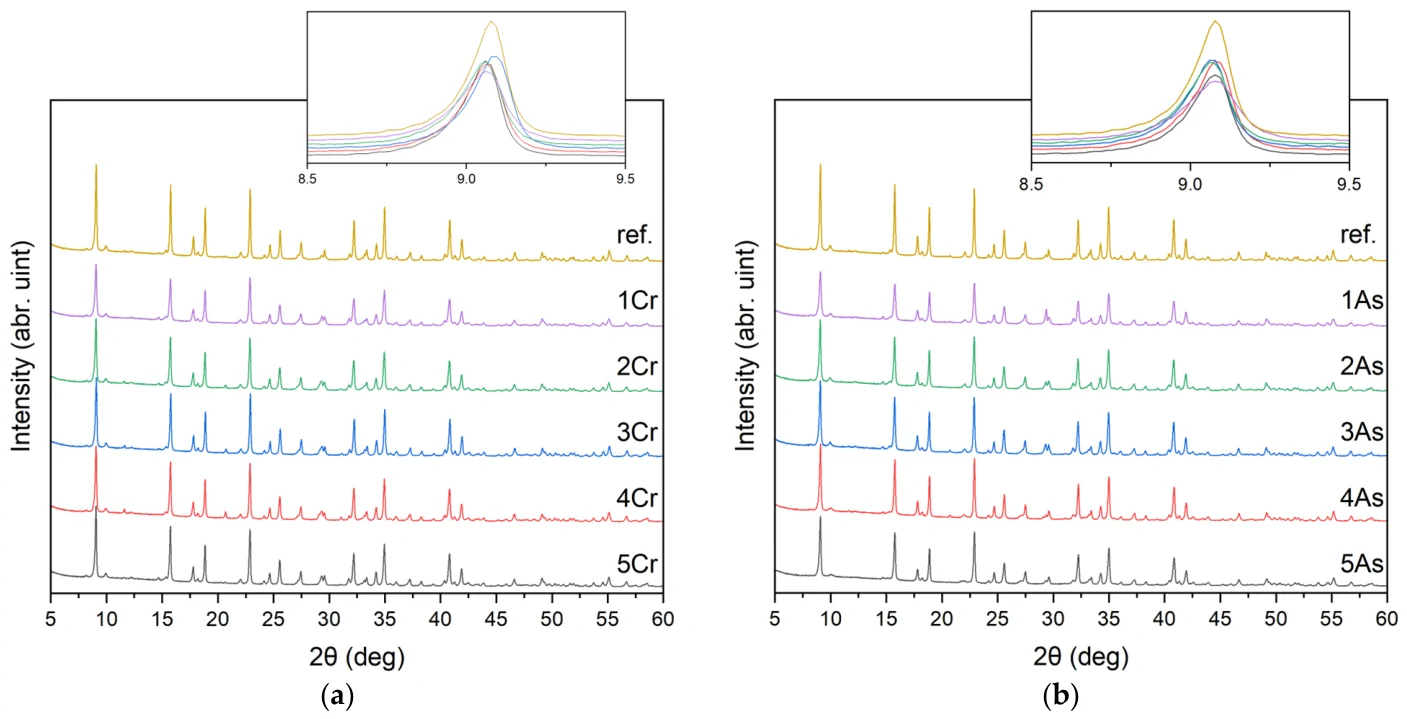
X-ray diffraction (XRD) patterns of the reference ettringite and samples synthesized in the presence of (**a**) chromate(VI) and (**b**) arsenate(V) ions.

Figure 3 presents the X-ray diffraction patterns of the reference ettringite sample and samples synthesized in the presence of increasing amounts of chromate and arsenate ions. All diffraction patterns are characterized by sharp and well-defined reflections, indicating the formation of highly crystalline products. The diffraction profiles of all oxyanions-containing samples are very similar to that of the reference ettringite, with no substantial changes in the positions or relative intensities of the main reflections observed throughout the series; the small variations can be seen more clearly in the enlarged inset in Figure 3.

The characteristic diffraction peaks of ettringite are present in all samples, confirming that ettringite remained the dominant crystalline phase. No pronounced peak broadening or significant changes in peak shape were observed, suggesting that the overall crystallinity of the synthesized materials was preserved. Only minor variations in the diffraction peak positions can be observed for individual samples, whereas no systematic shift in the reflections towards higher or lower 2θ values is evident. No additional intense diffraction peaks attributable to secondary crystalline phases were detected within the investigated diffraction range. However, the absence of detectable diffraction peaks does not necessarily indicate the complete absence of a secondary phase, particularly when its concentration is low and may be below the detection limit of XRD. To quantify the subtle structural variations, the lattice parameters obtained from Rietveld refinement were further analysed (Table 2). The refined values reveal some variations in the lattice parameters and the corresponding unit cell volume among the samples. The chromate-containing samples exhibited only minor fluctuations in the refined lattice parameters, resulting in unit cell volumes within a narrow range of 2.35329–2.35650 nm^3^. In contrast, the arsenate-containing series showed a gradual decrease in both lattice parameters and unit cell volume with increasing arsenate content, with the lowest values recorded for samples 4As and 5As.

The relative crystallinity index (CI) also varied among the synthesized samples (Table 2). Compared with the reference ettringite (CI = 100%), all modified samples exhibited lower CI values. For both oxyanion series, the crystallinity index increased from the first to the intermediate samples, reaching a maximum of 84.1% for sample 3Cr and 82.6% for sample 4As, followed by a slight decrease for the samples synthesized with the highest oxyanion concentrations. The CI ranged from 66.0% to 84.1% in the Cr series and from 58.9% to 82.6% in the As series. In the latter, a decrease in crystallinity was observed for the sample with the highest arsenate addition (5As), suggesting that excessive arsenate concentrations may hinder the development of a well-ordered crystal structure.

**Figure 4:**
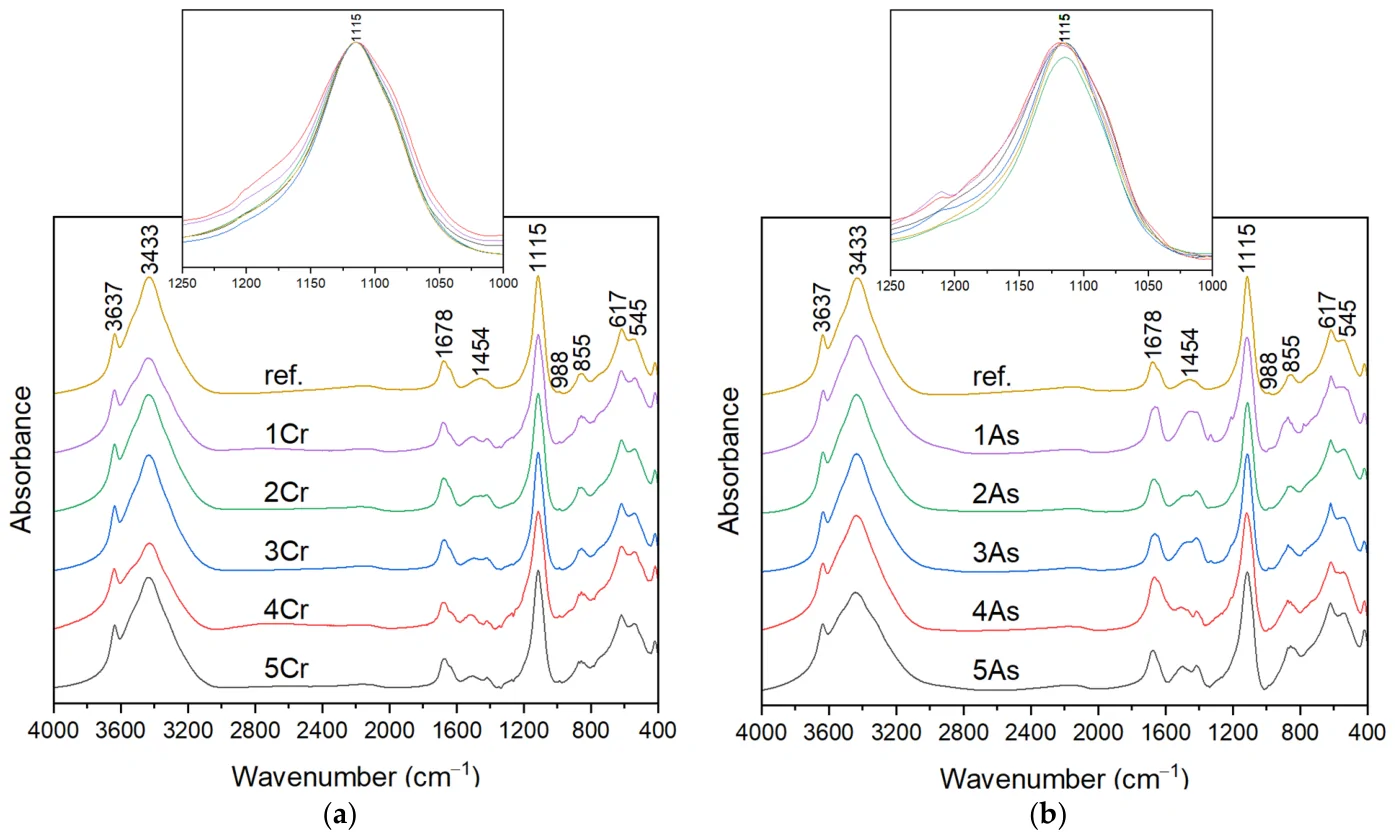
Fourier-transform infrared (FT-IR) spectra of the reference ettringite and samples synthesized in the presence of (**a**) chromate(VI) and (**b**) arsenate(V) ions.

Figure 4 presents the FT-IR spectra of the reference ettringite and samples synthesized in the presence of increasing amounts of chromate and arsenate ions. The FT-IR spectrum of the reference ettringite exhibits the characteristic absorption bands of ettringite, including a broad band in the 3700–3000 cm^−1^ region assigned to O–H stretching vibrations of structural hydroxyl groups and water molecules, a band at approximately 1678 cm^−1^ associated with H–O–H bending vibrations, and an intense band centred at about 1115 cm^−1^, attributed to the asymmetric stretching vibrations of S–O bonds in the sulfate groups. Additional absorption bands are observed in the low-wavenumber region, particularly at approximately 988, 855, 617 and 545 cm^−1^. The positions of the principal absorption bands identified in the FT-IR spectra and their assignments are summarized in Table 3.

The spectra of the chromate-containing samples are generally similar to that of the reference material, with all major absorption bands remaining present throughout the series. No substantial changes in the overall spectral profile are observed with increasing chromate addition. Only slight variations in the relative intensities and shapes of several absorption bands can be noticed.

Similarly, all arsenate-bearing samples exhibit the characteristic absorption bands of ettringite. However, compared with the chromate series, more pronounced changes in the relative intensities and band shapes are observed with increasing arsenate addition, particularly in the regions between approximately 1700–1400 cm^−1^ and 1200–800 cm^−1^. Despite these variations, the principal absorption bands characteristic of ettringite remain visible in all samples, indicating that the overall structural framework is preserved.

**Table 3:** Assignment of the characteristic FT-IR absorption bands observed for the reference ettringite and samples synthesized in the presence of chromate and arsenate ions [1,2,5,13].

Wavenumber, (cm^−1^)	Assignment	Remarks
418	δ(Al–OH)	ettringite framework
545	ν_3_(SO_4_^2−^) + ν(Al–O) vibrations of [AlO_6_]	overlapping vibrations
617	ν_4_(SO_4_^2−^)	possibly with contribution from δ(Al–OH)
855	δ(Al–OH) vibration of [AlO_4_]	may overlap with As–O vibrations
988	ν_1_(SO_4_^2−^)	sulfate group
1115	ν_3_(SO_4_^2−^)	sulfate group
1454	ν_3_(CO_3_^2−^)	carbonate species
1678	δ(H_2_O)	crystallization water and adsorbed moisture
3433	ν(O–H)	hydrogen-bonded water molecules
3637	ν(O–H)	structural OH groups not involved in hydrogen bonding

Considering the possible incorporation of oxyanions into the ettringite structure, substitution of sulfate groups by arsenate(V) or chromate(VI) may be associated with additional spectral features. Arsenate incorporation, expected in the form of HAsO_4_^2−^, may introduce As–O-related vibrations in the 1000–700 and 460–420 cm^−1^ regions. Due to overlap with characteristic ettringite bands, particularly δ(Al–OH) vibrations, these contributions may result in broadening or splitting of existing bands rather than the appearance of clearly separated peaks. The presence of HAsO_4_^2−^ may also affect the O–H stretching region due to changes in the hydrogen-bonding environment. For chromate-containing samples, additional ν(Cr–O) vibrations are expected in the region of approximately 880–900 cm^−1^. However, because of band overlap and the relatively low oxyanion content, such changes may remain difficult to resolve directly in the FT-IR spectra.

## 3. Discussion

The incorporation of chromate(VI) and arsenate(V) into the ettringite structure was investigated to determine how these oxyanions influence the structural stability and crystallographic features of the resulting phases. The combined results obtained from XRD, FT-IR, SEM, and XRF analyses indicate that the presence of both oxyanions does not prevent ettringite formation and that ettringite remains the dominant crystalline phase in all synthesized materials. However, the extent of structural modification depends strongly on the type and concentration of the incorporated oxyanion. While chromate(VI)-bearing samples preserved the structural characteristics of the reference ettringite with only minor changes in crystallographic parameters, arsenate(V) incorporation resulted in more pronounced modifications of the unit cell dimensions and vibrational features. These differences are discussed below in relation to the substitution mechanism, oxyanion characteristics, and the structural role of water molecules within the ettringite framework.

**Figure 5:**
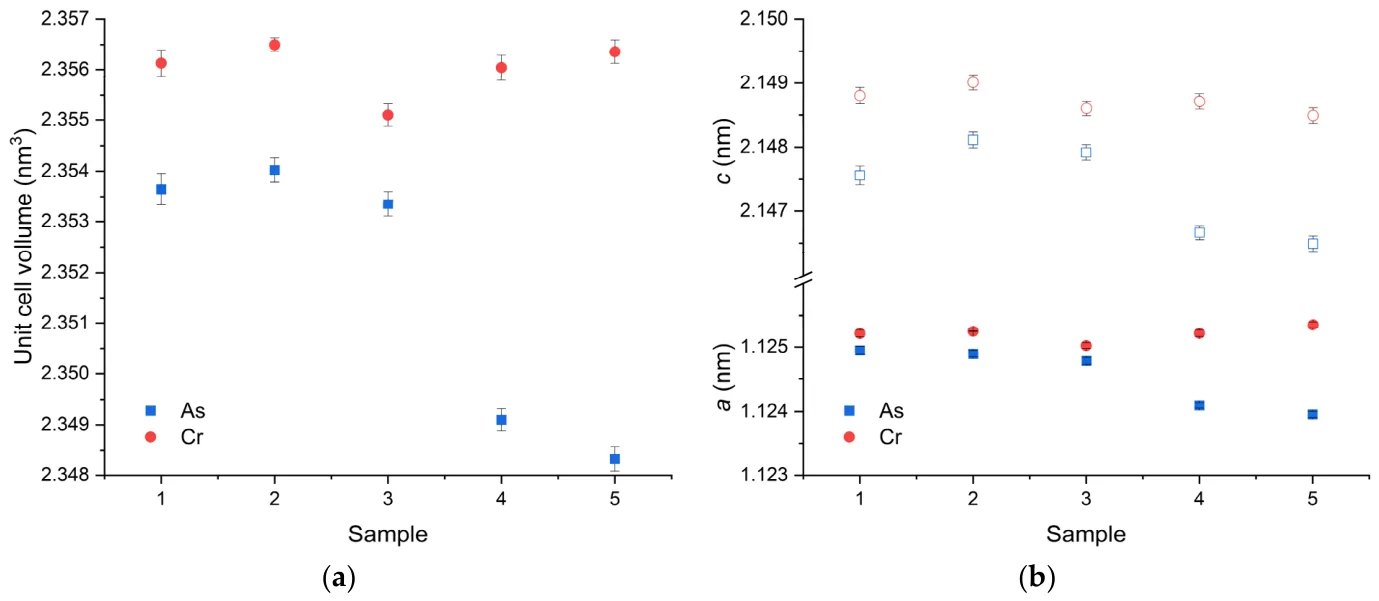
Evolution of unit cell volume (*V*) and lattice parameters (*a* and *c*) of ettringite as a function of the nominal oxyanion concentration: (**a**) unit cell volume and (**b**) lattice parameters *a* (filled symbols) and *c* (open symbols) for chromate- and arsenate-containing samples. Error bars represent the estimated standard deviations (ESDs) obtained from XRD refinement.

Figure 5a shows the variation in the unit cell volume of ettringite, determined from XRD refinement, as a function of increasing arsenate and chromate additions during synthesis. The unit cell volume of chromate-bearing ettringite showed only minor variations within the experimental uncertainty, indicating that increasing chromate concentration did not significantly affect the crystal lattice dimensions. Conversely, the unit cell volume of arsenate-bearing samples showed a non-monotonic variation with increasing arsenate addition. The most pronounced contraction was observed for samples 4As and 5As. The contrasting trends suggest that chromate incorporation induces only negligible structural distortions, whereas arsenate is associated with small changes in the ettringite unit-cell dimensions, particularly at higher arsenate concentrations.

Figure 5b shows the evolution of the lattice parameters *a* and *c*. The chromate-bearing samples exhibited only minor fluctuations in both lattice parameters, indicating that chromate incorporation had a negligible effect on the crystal lattice dimensions. In contrast, the arsenate-bearing samples displayed a non-monotonic decrease in both *a* and *c* with increasing arsenate addition, with the most pronounced changes observed for samples 4As and 5As. The reduction was particularly evident for the *c* parameter, suggesting that arsenate incorporation preferentially affected the structural features associated with the channels extending along the crystallographic *c*-axis. Consequently, the decrease in unit cell volume observed for the arsenate series resulted from the simultaneous contraction of both lattice parameters, dominated by the shortening of the *c* axis.

The XRF results presented in Table 1 show a progressive increase in the total chromium and arsenic content across both series. The total chromium content, expressed as Cr_2_O_3_ equivalent, increased from 0.02 wt.% in sample 1Cr to 1.31 wt.% in sample 5Cr, while the total arsenic content, expressed as As_2_O_5_ equivalent, increased from 0.13 wt.% in sample 1As to 9.01 wt.% in sample 5As. The higher arsenate content is consistent with the more pronounced changes in the unit cell dimensions observed for the arsenate-bearing samples, whereas the lower chromate content corresponds with the relatively minor changes observed in the chromate series.

In addition to the changes in the lattice parameters, the relative crystallinity index (CI) calculated from selected XRD peak intensities was also evaluated. The reference sample was assigned a CI value of 100%, whereas the oxyanion-containing samples showed lower CI values. However, because the CI is calculated from the relative intensities of the five most intense ettringite reflections, variations in this parameter may reflect changes in relative peak intensities and preferred orientation as well as differences in crystallinity. Therefore, the observed CI variations cannot be interpreted as direct evidence of changes in crystal ordering or crystallite development. The CI values varied non-monotonically across both oxyanion series, with no systematic concentration-dependent trend. Accordingly, the CI results are considered descriptive of the relative diffraction intensity of the investigated samples rather than as quantitative evidence of an oxyanion-induced effect on ettringite crystallization.

SEM observations (Figure 2) confirmed that the synthesized samples retained the characteristic acicular morphology of ettringite. Although some differences in crystal aggregation and development can be observed between the selected samples, these morphological features are not considered sufficient to establish a direct relationship with the variations in the crystallinity index. In particular, the arsenate-containing sample exhibited a more open network of well-developed needle-shaped crystals compared with the reference material. SEM observations primarily reflect crystal morphology and aggregation rather than structural changes at the atomic scale. Therefore, the morphological differences observed in the selected samples should be considered as an additional observation of crystal morphology rather than direct evidence of changes in the crystallinity index or crystal structure.

The decrease in unit cell volume observed for the arsenate-bearing samples is likely related primarily to the reduction in the *c* lattice parameter, indicating that arsenate incorporation preferentially affects structural features associated with the crystallographic *c* axis. The ettringite structure consists of positively charged Ca–Al hydroxide columns extending parallel to the *c* axis, with sulfate groups and water molecules located within the channels between these columns (Figure 1). Because of the large amount of structural water (~26 H_2_O per formula unit), the dimensions of these channels are strongly influenced by hydrogen-bonding interactions and the arrangement of water molecules within the structure. Therefore, the observed lattice contraction cannot be explained solely by differences in the size of the substituting oxyanion. Instead, it may reflect a more complex structural response associated with changes in the hydration environment of the ettringite channels.

Similar considerations have been reported for substituted ettringites, where incorporation of oxyanions into the sulfate positions was shown to modify the local structural arrangement, depending on the charge, geometry, and interaction of the substituting species with the surrounding hydration network. In the case of arsenate substitution, the replacement of SO_4_^2−^ by AsO_4_^3−^ requires charge compensation due to the difference in valence between these oxyanions. In [7], it was demonstrated that arsenate can be incorporated into ettringite channels as HAsO_4_^2−^, forming sulfate–arsenate ettringite solid solutions. Such substitution may induce local rearrangement of the surrounding Ca coordination environment and hydrogen-bonding interactions involving channel water molecules. Consequently, the structural channels may become more compact, resulting in contraction along the *c* axis and *a* decrease in the overall unit cell volume.

Similar structural responses have been reported for both synthetic AFt solid solutions and natural ettringite-group minerals [16,18]. Isovalent substitution (such as chromate for sulfate) generally results in linear unit-cell volume variations following Vegard’s law with minimal framework distortion [16,18]. In contrast, heterovalent substitution, such as incorporation of AsO_4_^3−^, requires charge compensation; in arsenate-bearing ettringite, this is achieved through protonation of the arsenate group to form HAsO_4_^2−^ [7]. Such substitution modifies the channel chemistry and associated hydrogen-bonding environment and can consequently produce anisotropic changes in the lattice parameters [7,19,20,21]. The present results are consistent with this structural interpretation. While arsenate-bearing samples exhibited a systematic decrease in the *c* parameter with increasing arsenate addition, with the most pronounced decrease observed for samples 4As and 5As. Chromate-bearing samples showed only minor variations in both lattice parameters. This difference may result from the distinct substitution mechanisms of the two oxyanions. Chromate substitution for sulfate is isovalent (CrO_4_^2−^ ↔ SO_4_^2−^). In contrast, arsenate incorporation introduces additional structural requirements related to charge compensation and therefore has a greater potential to modify the hydration environment of the ettringite framework.

Although changes in water content or water arrangement cannot be directly confirmed from X-ray diffraction data alone, the selective contraction of the *c* axis suggests that modification of the channel region, including possible rearrangement of structural water and hydrogen bonding, contributes to the observed structural response of arsenate-bearing ettringite. The selective contraction of the c-axis is consistent with literature reports regarding the perturbation of the channel region and its hydrogen-bonding network upon arsenate incorporation [5]. Therefore, the different structural responses observed for chromate- and arsenate-bearing ettringites may stem not only from substitution stoichiometry but also from distinct local interactions within the hydrated channels. Further structural studies, such as neutron diffraction, would be necessary to fully map these subtle hydrogen-bonding modifications.

The preservation of the characteristic acicular morphology indicates that neither chromate nor arsenate inhibited ettringite crystallization under the applied synthesis conditions. However, the more open crystal arrangement observed for the arsenate-bearing sample, together with the reduction in lattice parameters obtained from Rietveld refinement, suggests that arsenate affected crystal growth and aggregation to a greater extent than chromate. These observations are further supported by FT-IR analysis, discussed in the following section, which revealed more pronounced spectral modifications in the arsenate series.

The FT-IR spectra indicate that the overall ettringite framework remained preserved after synthesis in the presence of both oxyanions, as all characteristic absorption bands of ettringite were retained. However, subtle but systematic changes in band shape and relative intensity were observed, particularly for the samples synthesized with the highest arsenate concentration, suggesting that the local structural environment was modified despite the absence of major changes in the long-range crystal structure.

In the O–H stretching region (3700–3000 cm^−1^), a slight variation in band intensity and shape can be observed among the investigated samples. For sample 5As, a subtle decrease in the relative intensity of this band may indicate changes in the hydrogen-bonding environment and the arrangement of structural water associated with arsenate incorporation. However, a similar variation is also observed for sample 4Cr, indicating that this effect is not specific to arsenate. Therefore, the observed changes in the O–H stretching region should be interpreted cautiously and considered as a possible indication of changes in the local hydration environment rather than direct evidence of arsenate incorporation.

**Figure 6:**
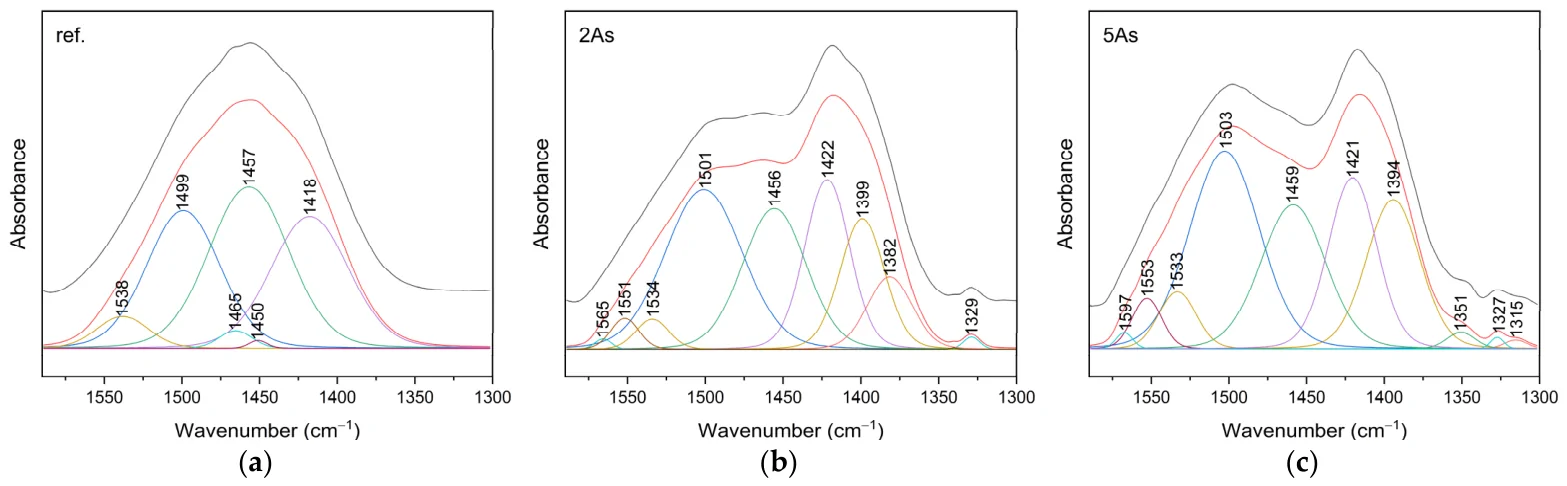
Deconvolution of the FT-IR absorption band in the 1590–1300 cm^−1^ region for (**a**) reference ettringite, (**b**) arsenate-bearing ettringite sample 2As, and (**c**) arsenate-bearing ettringite sample 5As. The experimental spectrum, fitted curve, and individual band components are shown.

More pronounced structural insights into the channel environment were obtained from 1500–1400 cm^−1^ spectral region (Figure 6). This region corresponds to the asymmetric stretching vibration of carbonate species (ν_3_ CO_3_^2−^), which originates from minor atmospheric CO_2_ absorption during synthesis/handling and trace carbonation of starting reagents. Although carbonate is considered a secondary species in the present samples, carbonate-related bands may provide indirect information on changes in the local chemical environment. However, the FT-IR data alone do not establish the crystallographic location of carbonate species within the ettringite structure. Such behaviour is consistent with a reduction in the local symmetry of carbonate environments caused by differences in hydrogen-bonding interactions and local coordination within the ettringite channels. While the reference ettringite exhibits a relatively simple band profile (Figure 6a), the incorporation of arsenate (sample 5As, Figure 6c) induces a distinct splitting and an increased complexity of analyzed mode. Band deconvolution further demonstrated that, while the reference ettringite spectrum could be adequately described using only a few carbonate-related components (Figure 6a), the spectrum of sample 5As required a considerably larger number of individual bands to reproduce the experimental profile (Figure 6c). The positions of the principal components remained nearly unchanged, indicating that the average carbonate environment was largely preserved. However, the appearance of additional low-intensity components and the increased band complexity indicate that the degeneracy of the ν_3_ carbonate vibration was partially lifted. Such behaviour may reflect differences in the local symmetry of carbonate environments including variations in hydrogen-bonding interactions and local coordination. However, the observed band splitting cannot be used as direct evidence for modification of the ettringite channel structure. Similar splitting of the ν_3_ carbonate band has been reported for partially carbonated ettringite and AFt phases, where carbonate species occupy environments of different symmetry following interaction with atmospheric CO_2_ [7,18]. In the arsenate-containing samples, particularly 5As, the increased complexity of the carbonate-related bands may reflect a more heterogeneous local environment. However, because a clear progressive trend is not observed across the arsenate series, this observation is considered only indirect and cannot be uniquely attributed to arsenate incorporation. Thus, the spectral changes observed in the arsenate series may result from the combined effects of partial carbonation and arsenate incorporation rather than from carbonation alone.

Because carbonate ions are smaller than sulfate ions, partial carbonate incorporation may also contribute to the slight contraction of the lattice parameters observed by XRD. Nevertheless, the absence of additional crystalline phases and the preservation of the characteristic ettringite bands suggest that carbonation, if present, represents only a secondary process. Instead, the increased band complexity is more likely associated with greater local structural heterogeneity within the ettringite channels, where carbonate, sulfate, structural water and incorporated oxyanions coexist and interact.

In contrast, the sulfate band centred at approximately 1115 cm^−1^ remained essentially unchanged throughout both sample series. Only very small shifts in band position, on the order of a few wavenumbers, were observed, which are comparable to the experimental uncertainty of the measurements. This indicates that the sulfate tetrahedra retained a similar average coordination environment despite the presence of chromate and arsenate during synthesis. Such behaviour is not unexpected, considering that the total chromium and arsenic contents determined by XRF (Table 1) were relatively low compared with the sulfate content of the ettringite structure. The relatively limited chromate concentration range investigated in the present study was selected according to the applied synthesis conditions and does not imply an upper limit for chromate incorporation into ettringite. Previous studies have demonstrated that ettringite-type structures can accommodate substantially higher chromate contents while retaining the characteristic framework [16,18]. Therefore, the minor crystallographic changes observed in the present chromate-bearing samples should be interpreted as a response within the investigated compositional range rather than as evidence of limited chromate incorporation capacity. Consequently, the vibrational contribution of sulfate remained dominant and any spectral changes associated with oxyanion substitution were largely masked by the intense sulfate absorption band. The relatively low total chromium and arsenic contents determined by XRF explains why their contribution to the FT-IR spectra remains subtle and requires detailed spectral analysis to be resolved. Thus, the absence of pronounced changes in the sulfate band does not exclude the presence of oxyanion-bearing species, but rather reflects the dominance of sulfate-related vibrations within the ettringite structure.

**Figure 7:**
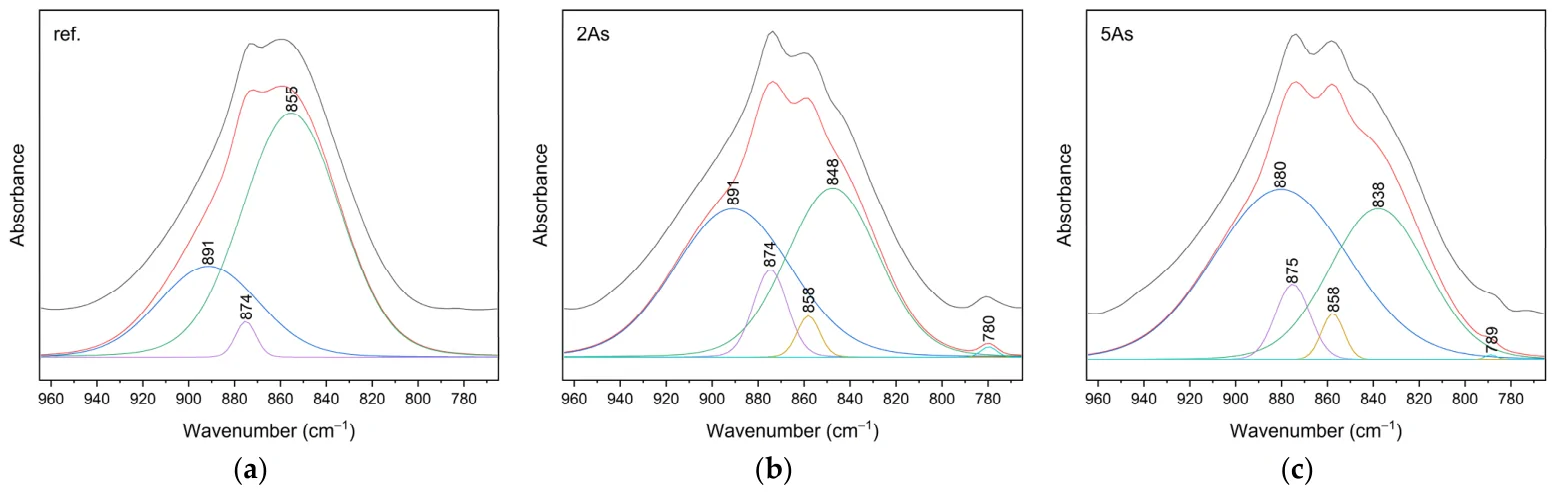
Deconvolution of the FT-IR absorption band in the 965–765 cm^−1^ region for (**a**) reference ettringite and (**b**) arsenate-bearing ettringite sample 2As, and (**c**) arsenate-bearing ettringite sample 5As. The experimental spectrum, fitted curve, and individual band components are shown.

To obtain more detailed information on the subtle spectral changes associated with arsenate incorporation, the complex absorption band in the 765–965 cm^−1^ region was subjected to band deconvolution (Figure 7). While the spectrum of the reference ettringite could be satisfactorily described using a limited number of components, the spectrum of sample 5As required an additional band centred at approximately 857 cm^−1^ to achieve an acceptable fit. Notably, an absorption band at approximately 855 cm^−1^ has previously been assigned to the As–O stretching vibration of HAsO_4_^2–^ in arsenate-bearing ettringite [7]. The band resolved at approximately 857 cm^−1^ in the present study is therefore consistent with the presence of arsenate-containing species. However, this spectral feature alone does not establish that the arsenic detected by XRF is incorporated into the ettringite sulfate sites or exclude its partial association with amorphous or poorly crystalline phases. The close agreement with the literature value supports the assignment of this component to an As–O vibration, but the FT-IR data do not allow the location or quantitative distribution of the arsenic species within the synthesized solids to be determined. At the same time, the fact that this feature becomes distinguishable only after spectral deconvolution suggests that the As–O vibration is largely masked by overlapping ettringite bands. In contrast, comparable spectral changes were not observed for the chromate-bearing samples, which is consistent with the smaller influence of chromate incorporation on the crystallographic parameters determined by XRD. This difference may be related to the distinct substitution mechanisms of the two oxyanions: chromate substitution for sulfate is isovalent, whereas incorporation of arsenate species, most likely in the form of HAsO_4_^2–^, involves additional charge-balancing requirements and may therefore induce stronger local rearrangement of the hydration environment.

The obtained results indicate that arsenate(V) and chromate(VI) are retained in the synthesized ettringite-bearing solids. However, the present data do not allow the fraction of the total arsenic and chromium content incorporated into the ettringite framework to be quantified or distinguished from possible retention in amorphous or poorly crystalline phases. The different structural responses observed for the two oxyanions indicate that their incorporation mechanism is strongly influenced by their chemical characteristics. While chromate(VI) can substitute for sulfate with only minor structural disturbance due to its isovalent character, arsenate(V) induces greater local rearrangement, likely related to its different charge distribution and interactions with the hydrogen-bonding network of structural water. Thus, ettringite is capable of accommodating both oxyanions while preserving its structural integrity.

Future research should focus on further elucidating the local coordination environment of incorporated oxyanions and distinguishing the contribution of different retention mechanisms. Advanced spectroscopic techniques, such as X-ray absorption spectroscopy, could provide direct information on the bonding environment and oxidation state of arsenic and chromium within the ettringite structure. In addition, long-term ageing and leaching studies would be valuable to evaluate the stability of oxyanion-containing ettringite under conditions relevant to environmental applications. Further investigations should also clarify the possible contribution of secondary processes, such as carbonation, to the structural evolution of substituted ettringite phases.

## 4. Materials and Methods

### 4.1. Sample Preparation

Synthetic ettringite was prepared according to the method proposed by Odler and Abdul-Maul [13,22,23]. A suspension of aluminum sulfate octadecahydrate with a concentration of 0.02 mol·L^−1^ and calcium hydroxide with a concentration of 0.12 mol·L^−1^ was prepared in 200 mL beakers. After mixing the reagents, demineralized water with a conductivity of 0.05 μS·cm^−1^ was added. The suspension was stirred at room temperature using a laboratory stirrer at 1400 rpm for 48 h. The resulting precipitate was separated by filtration, washed with acetone, and dried at room temperature. The synthesis step was repeated twice, yielding products with identical chemical and phase compositions, confirming the reproducibility of the procedure.

Subsequently, solid solutions of ettringite containing arsenate(V) and chromate(VI) oxyanions were synthesized. The samples were prepared analogously to the reference ettringite by mixing aluminum sulfate octadecahydrate and calcium hydroxide suspensions. For arsenate-modified samples, disodium hydrogen arsenate heptahydrate was used, whereas sodium chromate was applied for chromate-containing systems. The concentrations of oxyanions were 0.1, 0.5, 1, 5, and 10 mmol·L^−1^. This dosage range was selected to reflect environmentally relevant contamination levels in S/S applications while remaining within the solid-solution stability field of ettringite, avoiding the formation of secondary oxyanion precipitates. The suspensions were stirred at room temperature at 1000 rpm for 48 h while maintaining the pH in the range of 11.0–11.2 by controlled addition of hydrochloric acid solution. The obtained precipitates were filtered, washed with acetone, and dried at room temperature. The synthesis was repeated twice. The arsenate-containing samples were designated as 1As–5As, whereas the chromate-containing samples were labeled 1Cr–5Cr, according to the increasing concentration of introduced oxyanions.

All synthesized samples were subjected to structural characterization, including X-ray diffraction (XRD), Fourier-transform infrared spectroscopy (FT-IR), scanning electron microscopy (SEM), and elemental composition analysis (XRF).

### 4.2. Characterization Methods

The elemental composition of the synthesized samples was determined by wavelength-dispersive X-ray fluorescence spectroscopy (WD-XRF). The measurements were performed using an Axios Max spectrometer (PANalytical, Malvern, UK) equipped with a 4 kW Rh anode X-ray tube. The obtained spectra were processed using the Omnian software package (PANalytical, Malvern, UK), allowing determination of the elemental composition of the synthesized ettringite-based materials.

The morphology of the synthesized materials was investigated using scanning electron microscopy (SEM). Prior to SEM analysis, the powdered samples were coated with a thin conductive gold layer by sputtering. The microstructural observations were performed in backscattered electron (BSE) mode using a Phenom XL desktop SEM (Thermo Fisher Scientific, Waltham, MA, USA) operated at an accelerating voltage of 15 kV.

The phase composition and structural characteristics of the synthesized materials were investigated by X-ray diffraction (XRD). The measurements were performed using a Philips X’Pert diffractometer (PANalytical, Malvern, UK) equipped with CuKα radiation (λ = 1.5418 Å). Diffraction data were collected over the 2θ range of 5–90° using a step size of 0.007°, with an acquisition time of approximately 2 h per sample. The recorded diffraction patterns were analyzed using Match! 4 software, and phase identification was carried out based on reference patterns available in the Crystallography Open Database (COD). The crystal structure parameters of the ettringite phase were determined by Rietveld refinement of the XRD patterns. The refinement was performed by least-squares minimization of the difference between the experimental and calculated diffraction profiles. The quality of the fit was assessed based on the reduced chi-square (chi^2^)) value and visual agreement between the experimental and calculated profiles. The refinement was considered converged when further refinement cycles did not result in significant improvement of the fit. The unit-cell parameters *a* and *c*, together with their estimated standard deviations (ESDs), were obtained for each sample and collected in Table 2. The unit cell parameters *a* and *c* were obtained for each sample, and the unit cell volume was calculated according to the hexagonal crystal system (1):

(1) $$V\;=\;\frac{\sqrt{3}}{2}a^{2}c,$$

where *a* and *c* correspond to the refined lattice parameters. The estimated standard deviations (ESDs) obtained during the refinement procedure were used to assess the uncertainty associated with the calculated values.

The relative crystallinity of the synthesized samples was evaluated based on the intensities of the five most intense ettringite reflections. The crystallinity index (*CI*) was calculated by comparing the sum of the selected peak intensities of each investigated sample with the corresponding value obtained for the reference ettringite sample, which was assigned a relative crystallinity value of 100% (2):

(2) $$CI(\%)=\frac{\sum I_{sample}}{\sum I_{ref.}}\times 100,$$

where $\sum I_{sample}$ represents the sum of the intensities of the selected ettringite reflections for the analyzed sample, and $\sum I_{ref.}$ corresponds to the sum of the intensities of the same reflections measured for the reference ettringite sample.

Mid-infrared (MIR) spectra were recorded using a Bruker VERTEX 70v Fourier-transform infrared spectrometer (Bruker, Billerica, MA, USA) under vacuum conditions. The residual pressure during the FT-IR measurements was below 0.1 hPa. Spectra were obtained in the range of 4000–400 cm^−1^ in absorbance mode using the conventional KBr pellet method (mg of the powdered sample was thoroughly mixed with 350 mg of KBr). Each spectrum represented the average of 128 scans at a spectral resolution of 4 cm^−1^. Before analysis, the spectra were baseline corrected and selected absorption regions were subjected to band deconvolution. Spectral decomposition was performed using the Fityk software package (1.3.1 version) [24]. The band-fitting procedure was carried out using mixed Gaussian-Lorentzian functions (90% Gaussian and 10% Lorentzian contribution). The number of fitted components was minimized while maintaining an acceptable agreement between the experimental and calculated spectra. Initial band positions were determined from the second derivative analysis of smoothed spectra, followed by final refinement with only minor adjustments of the band positions to maintain their physical meaning. Spectral smoothing was performed using the moving average method, with the smoothing parameters optimized according to the analysed spectral region.

## 5. Conclusions

Ettringite-based materials containing arsenate(V) and chromate(VI) were successfully synthesized, with ettringite remaining the dominant crystalline phase in all investigated samples. XRD analysis showed that ettringite remained the dominant phase in all samples, with no detectable secondary crystalline phases within the detection limits of the applied XRD method. However, arsenate incorporation resulted in more pronounced changes in lattice parameters, particularly a contraction of the unit cell volume, whereas chromate incorporation caused only minor structural variations.

FT-IR analysis confirmed the preservation of the characteristic ettringite vibrational features and revealed subtle modifications of the local structural environment. The deconvolution of selected absorption bands enabled the identification of an additional component at approximately 857 cm^−1^ in the highly arsenate-containing sample, consistent with the presence of arsenate-containing species in the highly arsenate-containing sample and with previous assignments of As–O vibrations in arsenate-bearing ettringite. However, the present XRF, XRD and FT-IR data do not allow the quantitative fraction of arsenic incorporated into the ettringite structure to be determined. The observed differences between arsenate- and chromate-bearing samples indicate that the extent of structural modification depends on the nature of the incorporated oxyanion and its interaction with the hydration environment of the ettringite channels.

## Figures and Tables

**Table 2:** Crystallographic parameters and Rietveld refinement statistics and crystallinity index (CI) of ettringite synthesized in the presence of increasing additions of arsenate and chromate ions *.

Sample Name	*a*, (nm)	*c*, (nm)	V, (nm^3^)	CI, (%)
ref.	1.124083(68)	2.14846(14)	2.35101(32)	100.0
1Cr	1.125217(53)	2.14880(13)	2.35613(26)	66.0
2Cr	1.125250(05)	2.14901(12)	2.35650(13)	74.0
3Cr	1.125023(45)	2.14860(11)	2.35510(22)	84.1
4Cr	1.125219(50)	2.14871(12)	2.35604(25)	78.9
5Cr	1.125353(47)	2.14849(12)	2.35636(24)	80.2
1As	1.124948(63)	2.14756(15)	2.35364(31)	58.9
2As	1.124895(48)	2.14811(12)	2.35403(24)	74.5
3As	1.124784(49)	2.14792(12)	2.35335(24)	79.9
4As	1.124097(44)	2.14666(11)	2.34910(22)	82.6
5As	1.123956(49)	2.14649(12)	2.34832(24)	72.1

* Estimated standard deviations (ESDs) are given in parentheses.

## Data Availability

The original contributions presented in this study are included in the article. Further inquiries can be directed to the corresponding author.

## Abbreviations

The following abbreviations are used in this manuscript:

AFm	aluminate–ferrite–monosulfate
AFt	aluminate–ferrite–trisulfate; ettringite
CI	crystallinity index
ESD	estimated standard deviation
FT-IR	Fourier-transform infrared spectroscopy
SEM	scanning electron microscopy
S/S	solidification/stabilization
XRD	X-ray diffraction
XRF	X-ray fluorescence
